# Lithotomy

**Published:** 1898-02

**Authors:** F. Paschal

**Affiliations:** San Antonio, Texas


### TEXAS MEDICAL JOURNAL.

ESTABLISHED JULY, 1885.

PUBLISHED MONTHLY.—SUBSCRIPTION $2.00 A YEAR.

VOL. XIII. AUSTIN, FEBRUARY, 1898. No. 8.

### Original Contributions.

For the Texas Medical Journal.

### LITHOTOMY.

BY F. PASCHAL, M. D., SAN ANTONIO, TEXAS.

[Read before the West Texas Medical Society.]

It is hardly to be supposed that I can offer anything new on the subject of stone and lithotomy; volumes have been written and history will no doubt have much more to say in regard to this matter.

The tendency of this age is to look for new "gods" in medicine and surgery, and while seeking them it may not be amiss for us to study the past, and convince ourselves that many of the new gods we are now worshipping are really not old ones long since relegated to obscurity, but are now brought forth dressed in new robes and held up to us for admiration and emulation. That our ancestors in medicine and surgery were very intelligent cannot for a moment be denied, for history is resplendent with their noble achievements; and although we may boast of more elegant means at our command to make our results better than their results, the fact remains that we, in this progressive age of medicine and surgery, are willing to take, so to speak, retrogressive steps, and follow the paths our ancestors emblazoned and abandoned, and we by virtue of better surroundings that education and advanced civilization have endowed us with, try to induce the profession to believe that old-fashioned operations and remedies failed our ancestors, but are after all the best, and will not fail us, because we are richer in knowledge, richer in purse and better equipped to perfect them. We should remember that pride sometimes goes before a fall, and that posterity will write our history and may place on the head lines thereof: "Failed to accomplish any more than their ancestors because they were no wiser or any better physicians and surgeons."

It is as important for the surgeon to know the cause of the formation and composition of stones found in the bladder and kidneys, as it is for him to know how and when to operate; for in removing a stone we only rid the patient of a foreign body that is likely to be reproduced unless proper means are employed to prevent its recurrence.

Stones have for their base organic and inorganic substances. Those having the organic base are the most frequent, and are classed as uric acid, urates, and their modifications, the oxalates, uric oxide and cystic oxide. The inorganic stones are composed principally of phosphates of lime, magnesia and ammonia. Then there are those rare varieties, the carbonate of lime, the fibrinous and uro-stetath.

The primary cause of the formation of stones having an organic base is supposed to be due to the so-called improper combustion, and improper assimilation of food. The peculiar vice of constitution that is supposed to give rise to this is thought to be heriditary or acquired, and by some means or other causes an increased amount of uric and oxalic salts to be eliminated and dissolved in the urine, and which becomes crystalized and precipitated in the kidneys or bladder. In the organic stones the formation of the stone begins in the kidneys (as a rule, from whence a small aggregation of these crystals start, a microscopic stone, as it were, so small is it at times), and passes into the bladder, where it is retained and causes catarrhal inflammation of the coats of the bladder, which in turn gather up particle after particle and cements them together, until stones are formed, varying from a few grains in weight, to six pounds, three ounces, and in number from a single stone to one thousand. Phosphatic stones are usually found in the bladder alone, and are due to decomposition of the urine, caused by disease of the coats of the bladder; and hence the bladder is the cause of the formation of this kind of stone, and the stone not the cause of the diseased bladder.

The cutting operation for removing stones dates back many ages, and has undergone modifications which marked the progress of surgery; and it would seem that even to this day we are trying to find the best route to the bladder. The two principal cutting methods for performing lithotomy are, the supra-pubic and the perineal. The superiority of one over the other is a matter of personal experience, the size of the stone, the age and condition of the patient determining it.

"The supra-pubic operation was first practiced in Paris in 1475 by Colat, as an experiment, on a criminal by permission of Louis XI, and the patient recovered in a fortnight. The earliest account of this method was published in 1556 by Pierre Franco. He performed it on a child two years old, after finding the calcules too large to be removed through the perineum." His remarks then tended to discourage the practice.

The perineal method is the most ancient, and was practiced upwards of two thousand years ago by Ammonius, at Alexandria, in the time of Herophilus, and by Meges in Rome during the reign of Augustus. It was known then as cutting on the gripe, and was done by introducing two fingers into the rectum, gripping the stone and pressing it against the perineum, where it was cut upon and removed. It was really the operation of Celsus or the operation of the apparatus minor.

The lateral operation remains today pretty much the same as it was in 1697, when Jacques did the operation with a sound, having cut, it is said, five thousand times; but the credit of perfecting the operation is usually awarded to Cheselden. Upon investigation it was found that Jacques, Como, Dougias, Cheselden, Paul, Pye, Molgil, Thombill and Souberville abandoned the supra-pubic operation on account of the large mortality, which amounted to one death for every $4\frac{8}{18}$ cases cut, and accepting the lateral operation, which gave a mortality of one in every eight cases operated upon. The results of these operations could not have been due to inferior methods of operating, for since the introduction of antiseptics or the aseptic age, the mortality is almost as proportionally great. Statistics show that since 1878 the supra-pubic operation gives a death rate in children under 14 years of age of $12\frac{52}{100}$, and for the same age by the perineal method a death rate of $2\frac{96}{100}$; adults from 14 to 50, by supra-pubic method, $12\frac{1}{100}$; by perineal method, $11\frac{36}{100}$; old men over 50 years of age, $32\frac{07}{100}$ by supra-pubic, and $16\frac{66}{100}$ by perineal method.

After comparing the death rate of the two operations in olden times and in our advanced age of surgical progress, what object is there for us to go back to the old method long since abandoned because of its larger mortality? As to the claim that it is easier of execution, traverses no important structures, and leaves no bad consequences, the fact remains that the death rate is greater in the supra-pubic operation, and fistulæ more frequently result, and it is really no easier of performance to a surgeon having ordinary skill.

As to impotency following the lateral operation, it is hardly worthy of serious consideration. In the lateral operation the prostate is divided on the left side, and as the duct penetrates the lower part of the substance of the prostate gland in order to reach the urethra, and as the knife divides the side of that gland obliquely inwards and upwards, or outwards and downwards, the seminal duct will not be in danger; and even should it be cut, the duct on the right side will remain uninjured. If the rectum is wounded low down, it is of no great importance; and with ordinary care it should not be wounded high up.

In conclusion, I will say that if my experience is worth anything I am willing to continue the lateral operation, having had two deaths in forty cases operated upon; and if the supra-pubic method of lithotomy proves to give a lower rate of mortality I will gladly accept it.

